# Bis(μ-2-methyl­quinolin-1-ium-8-olato-κ^2^ *O*:*O*′)bis­[(2-methyl­quinolin-1-ium-8-olato-κ*O*)tris­(nitrato-κ^2^ *O*,*O*′)lanthanum(III)]

**DOI:** 10.1107/S1600536809019746

**Published:** 2009-06-06

**Authors:** Yousef Fazaeli, Ezzatollah Najafi, Mostafa M. Amini, Seik Weng Ng

**Affiliations:** a Department of Chemistry, General Campus, Shahid Beheshti University, Tehran 1983963113, Iran; b Department of Chemistry, University of Malaya, 50603 Kuala Lumpur, Malaysia

## Abstract

The two independent *N*-heterocycles in the centrosymmetric title compound, [La_2_(C_10_H_9_NO)_4_(NO_3_)_6_], exist in the zwitterionic form. One of these binds to one metal center, whereas the other bridges two metal centers. The La atom is chelated by three nitrate groups and is surrounded by nine O atoms in a coordination environment based on a distorted monocapped square-anti­prism. The dinuclear structure is further stabilized by intra­molecular N—H⋯O(nitrate) hydrogen bonds.

## Related literature

The *N*-heterocycle exists in the deprotonated and neutral form in hexa­kis(*μ*-2-methyl­quinolin-8-oxido)bis­(2-methyl­quinolin-8-oxido(2-methyl-8-quinolinol)(nitrato)trilanthanum meth­an­ol solvate; see: Katkova *et al.* (2005 ▶).
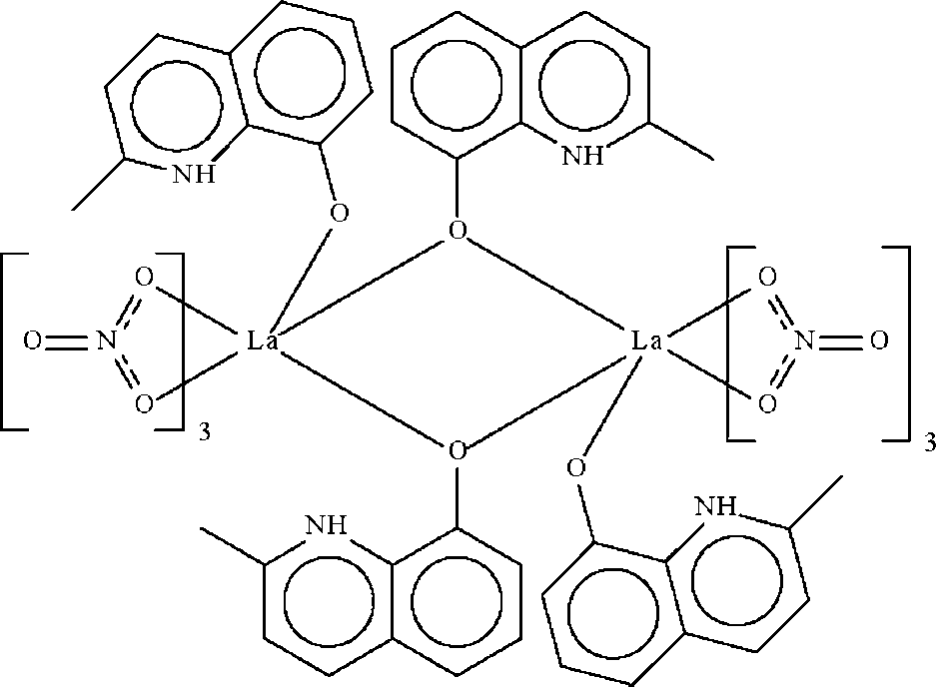


## Experimental

### 

#### Crystal data

[La_2_(C_10_H_9_NO)_4_(NO_3_)_6_]
*M* *_r_* = 1286.61
Monoclinic, 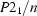

*a* = 10.7177 (2) Å
*b* = 18.3308 (3) Å
*c* = 12.4473 (2) Å
β = 109.952 (1)°
*V* = 2298.67 (7) Å^3^
*Z* = 2
Mo *K*α radiation
μ = 1.93 mm^−1^
*T* = 100 K
0.20 × 0.15 × 0.10 mm

#### Data collection

Bruker SMART APEX diffractometer
Absorption correction: multi-scan (*SADABS*; Sheldrick, 1996 ▶) *T*_min_ = 0.699, *T*_max_ = 0.830
28922 measured reflections
5258 independent reflections
4897 reflections with *I* > 2σ(*I*)
*R*_int_ = 0.020

#### Refinement

*R*[*F* ^2^ > 2σ(*F* ^2^)] = 0.022
*wR*(*F* ^2^) = 0.057
*S* = 1.05
5258 reflections
344 parameters
2 restraints
H atoms treated by a mixture of independent and constrained refinement
Δρ_max_ = 0.64 e Å^−3^
Δρ_min_ = −0.36 e Å^−3^

### 

Data collection: *APEX2* (Bruker, 2008 ▶); cell refinement: *SAINT* (Bruker, 2008 ▶); data reduction: *SAINT*; program(s) used to solve structure: *SHELXS97* (Sheldrick, 2008 ▶); program(s) used to refine structure: *SHELXL97* (Sheldrick, 2008 ▶); molecular graphics: *X-SEED* (Barbour, 2001 ▶); software used to prepare material for publication: *publCIF* (Westrip, 2009 ▶).

## Supplementary Material

Crystal structure: contains datablocks global, I. DOI: 10.1107/S1600536809019746/tk2454sup1.cif

Structure factors: contains datablocks I. DOI: 10.1107/S1600536809019746/tk2454Isup2.hkl

Additional supplementary materials:  crystallographic information; 3D view; checkCIF report

## Figures and Tables

**Table 1:** Hydrogen-bond geometry (Å, °)

*D*—H⋯*A*	*D*—H	H⋯*A*	*D*⋯*A*	*D*—H⋯*A*
N1—H1⋯O4^i^	0.88 (1)	2.39 (2)	3.115 (3)	140 (2)
N2—H2⋯O3	0.87 (1)	2.08 (1)	2.950 (2)	173 (2)

Symmetry code: (i) 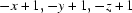.

**Fig. 1.**
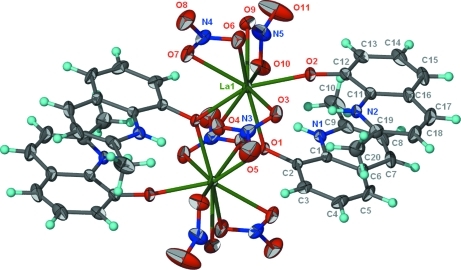
Thermal ellipsoid plot (Barbour, 2001) of [La(NO3)3(C10H9NO)2]2; ellipsoids are drawn at the 70% probability level and H atoms of arbitrary radius.

**Fig. 2.**
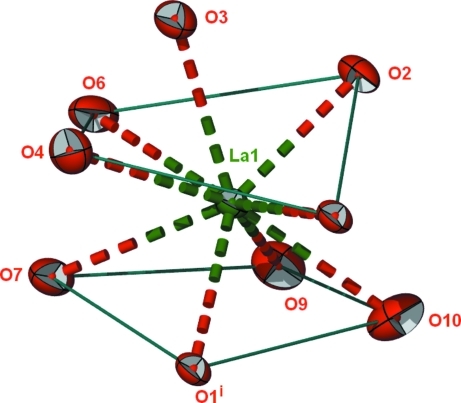
Monocapped square-antiprismatic geometry of La.

## supplementary crystallographic information

### Experimental

2-Methyl-8-hydroxyquinoline (0.32 g, 2 mmol) was added to lanthanum nitrate hexahydrate (0.43 g, 1 mmol) in methanol (10 ml). The mixture was stirred for an hour and then filtered. Slow evaporation of solution gave yellow crystals that are stable when heated up to 573 K.

### Refinement

Carbon-bound H-atoms were placed in calculated positions (C—H 0.95 to 0.98 Å) and were included in the refinement in the riding model approximation, with *U*(H) set to 1.2 to 1.5*U*(C). The nitrogen-bound hydrogen atoms were located in a difference Fourier map, and were refined with a distance restraint of N–H 0.88±01 Å; their temperature factors were freely refined.

### Crystal data

**Table:**

[La_2_(C_10_H_9_NO)_4_(NO_3_)_6_]	*F*(000) = 1272
*M**_r_* = 1286.61	*D*_x_ = 1.859 Mg m^−^^3^
Monoclinic, *P*2_1_/*n*	Mo *K*α radiation, λ = 0.71073 Å
Hall symbol: -P 2yn	Cell parameters from 9940 reflections
*a* = 10.7177 (2) Å	θ = 2.3–28.3°
*b* = 18.3308 (3) Å	µ = 1.93 mm^−^^1^
*c* = 12.4473 (2) Å	*T* = 100 K
β = 109.952 (1)°	Block, yellow
*V* = 2298.67 (7) Å^3^	0.20 × 0.15 × 0.10 mm
*Z* = 2	

### Data collection

**Table:**

Bruker SMART APEX diffractometer	5258 independent reflections
Radiation source: fine-focus sealed tube	4897 reflections with *I* > 2σ(*I*)
graphite	*R*_int_ = 0.020
ω scans	θ_max_ = 27.5°, θ_min_ = 2.1°
Absorption correction: multi-scan (*SADABS*; Sheldrick, 1996)	*h* = −13→13
*T*_min_ = 0.699, *T*_max_ = 0.830	*k* = −23→23
28922 measured reflections	*l* = −16→15

### Refinement

**Table:**

Refinement on *F*^2^	Primary atom site location: structure-invariant direct methods
Least-squares matrix: full	Secondary atom site location: difference Fourier map
*R*[*F*^2^ > 2σ(*F*^2^)] = 0.022	Hydrogen site location: inferred from neighbouring sites
*wR*(*F*^2^) = 0.057	H atoms treated by a mixture of independent and constrained refinement
*S* = 1.05	*w* = 1/[σ^2^(*F*_o_^2^) + (0.0293*P*)^2^ + 2.866*P*] where *P* = (*F*_o_^2^ + 2*F*_c_^2^)/3
5258 reflections	(Δ/σ)_max_ = 0.001
344 parameters	Δρ_max_ = 0.64 e Å^−^^3^
2 restraints	Δρ_min_ = −0.36 e Å^−^^3^

### Fractional atomic coordinates and isotropic or equivalent isotropic displacement parameters (Å^2^)

**Table:**

	*x*	*y*	*z*	*U*_iso_*/*U*_eq_	
La1	0.627521 (11)	0.471863 (6)	0.661979 (10)	0.01366 (5)	
O1	0.39482 (14)	0.47330 (8)	0.52498 (13)	0.0153 (3)	
O2	0.55263 (15)	0.38409 (8)	0.76113 (14)	0.0197 (3)	
O3	0.51647 (17)	0.53810 (9)	0.79000 (15)	0.0241 (3)	
O4	0.57975 (17)	0.61199 (9)	0.68318 (15)	0.0259 (4)	
O5	0.4652 (2)	0.65310 (11)	0.7854 (2)	0.0433 (5)	
O6	0.81044 (17)	0.50974 (11)	0.84912 (14)	0.0281 (4)	
O7	0.84706 (16)	0.54647 (10)	0.69668 (14)	0.0249 (3)	
O8	0.97394 (18)	0.58366 (12)	0.86437 (17)	0.0380 (5)	
O9	0.81082 (16)	0.37627 (10)	0.69806 (16)	0.0287 (4)	
O10	0.63565 (19)	0.34935 (10)	0.55359 (15)	0.0311 (4)	
O11	0.7770 (3)	0.26474 (13)	0.6382 (2)	0.0668 (8)	
N1	0.31296 (18)	0.33149 (10)	0.50479 (16)	0.0185 (4)	
H1	0.379 (2)	0.3456 (15)	0.483 (2)	0.026 (7)*	
N2	0.37563 (17)	0.42049 (11)	0.86348 (15)	0.0178 (4)	
H2	0.423 (2)	0.4525 (11)	0.842 (2)	0.017 (6)*	
N3	0.51907 (19)	0.60337 (11)	0.75377 (18)	0.0234 (4)	
N4	0.87999 (19)	0.54744 (12)	0.80494 (18)	0.0234 (4)	
N5	0.7429 (2)	0.32815 (12)	0.62974 (19)	0.0313 (5)	
C1	0.2499 (2)	0.38214 (12)	0.54963 (17)	0.0156 (4)	
C2	0.2910 (2)	0.45585 (12)	0.55661 (17)	0.0151 (4)	
C3	0.2200 (2)	0.50601 (13)	0.59584 (19)	0.0201 (4)	
H3	0.2432	0.5562	0.5991	0.024*	
C4	0.1140 (2)	0.48398 (15)	0.6309 (2)	0.0255 (5)	
H4	0.0661	0.5197	0.6563	0.031*	
C5	0.0788 (2)	0.41235 (15)	0.6292 (2)	0.0263 (5)	
H5	0.0089	0.3982	0.6555	0.032*	
C6	0.1471 (2)	0.35933 (13)	0.58808 (18)	0.0211 (4)	
C7	0.1194 (2)	0.28332 (14)	0.5841 (2)	0.0281 (5)	
H7	0.0524	0.2656	0.6114	0.034*	
C8	0.1881 (3)	0.23603 (13)	0.5416 (2)	0.0290 (5)	
H8	0.1694	0.1853	0.5408	0.035*	
C9	0.2862 (2)	0.26032 (12)	0.49867 (19)	0.0235 (5)	
C10	0.3615 (3)	0.21221 (14)	0.4471 (2)	0.0340 (6)	
H10A	0.3016	0.1754	0.3991	0.051*	
H10B	0.4328	0.1879	0.5078	0.051*	
H10C	0.3997	0.2415	0.4001	0.051*	
C11	0.3925 (2)	0.34856 (12)	0.84219 (18)	0.0176 (4)	
C12	0.4870 (2)	0.33087 (12)	0.78824 (18)	0.0173 (4)	
C13	0.5007 (2)	0.25687 (12)	0.76861 (19)	0.0213 (4)	
H13	0.5619	0.2422	0.7327	0.026*	
C14	0.4262 (3)	0.20362 (13)	0.8008 (2)	0.0265 (5)	
H14	0.4391	0.1537	0.7868	0.032*	
C15	0.3352 (3)	0.22155 (14)	0.8518 (2)	0.0280 (5)	
H15	0.2859	0.1845	0.8727	0.034*	
C16	0.3157 (2)	0.29544 (13)	0.87294 (19)	0.0222 (5)	
C17	0.2211 (2)	0.32082 (15)	0.9205 (2)	0.0293 (5)	
H17	0.1653	0.2866	0.9396	0.035*	
C18	0.2081 (2)	0.39351 (15)	0.9397 (2)	0.0286 (5)	
H18	0.1446	0.4093	0.9725	0.034*	
C19	0.2892 (2)	0.44488 (14)	0.91067 (19)	0.0229 (5)	
C20	0.2841 (3)	0.52479 (14)	0.9304 (2)	0.0278 (5)	
H20A	0.3358	0.5506	0.8908	0.042*	
H20B	0.3216	0.5349	1.0125	0.042*	
H20C	0.1917	0.5414	0.9009	0.042*	

### Atomic displacement parameters (Å^2^)

**Table:**

	*U*^11^	*U*^22^	*U*^33^	*U*^12^	*U*^13^	*U*^23^
La1	0.01291 (7)	0.01585 (7)	0.01372 (7)	−0.00115 (4)	0.00648 (5)	0.00089 (4)
O1	0.0135 (7)	0.0175 (7)	0.0165 (7)	−0.0023 (5)	0.0073 (6)	0.0011 (6)
O2	0.0205 (7)	0.0183 (7)	0.0243 (8)	0.0005 (6)	0.0129 (6)	0.0048 (6)
O3	0.0275 (9)	0.0219 (8)	0.0293 (9)	−0.0028 (6)	0.0182 (7)	−0.0025 (7)
O4	0.0336 (9)	0.0189 (8)	0.0277 (9)	−0.0011 (7)	0.0135 (7)	0.0016 (7)
O5	0.0421 (11)	0.0277 (10)	0.0687 (15)	0.0063 (8)	0.0302 (11)	−0.0141 (10)
O6	0.0249 (9)	0.0433 (10)	0.0175 (8)	−0.0090 (8)	0.0092 (7)	−0.0023 (7)
O7	0.0203 (8)	0.0363 (9)	0.0202 (8)	−0.0081 (7)	0.0094 (6)	−0.0026 (7)
O8	0.0248 (9)	0.0518 (12)	0.0333 (10)	−0.0167 (8)	0.0045 (8)	−0.0121 (9)
O9	0.0206 (8)	0.0328 (9)	0.0354 (10)	0.0050 (7)	0.0129 (7)	0.0039 (8)
O10	0.0399 (10)	0.0305 (9)	0.0211 (9)	0.0092 (8)	0.0079 (8)	−0.0024 (7)
O11	0.105 (2)	0.0392 (13)	0.0404 (13)	0.0431 (14)	0.0049 (13)	−0.0060 (10)
N1	0.0187 (9)	0.0173 (9)	0.0174 (9)	−0.0025 (7)	0.0035 (7)	0.0021 (7)
N2	0.0171 (8)	0.0223 (9)	0.0157 (9)	−0.0016 (7)	0.0078 (7)	0.0035 (7)
N3	0.0192 (9)	0.0204 (9)	0.0304 (11)	0.0000 (7)	0.0083 (8)	−0.0055 (8)
N4	0.0171 (9)	0.0310 (10)	0.0226 (10)	−0.0028 (8)	0.0073 (8)	−0.0048 (8)
N5	0.0433 (13)	0.0308 (11)	0.0245 (11)	0.0161 (10)	0.0175 (10)	0.0032 (9)
C1	0.0131 (9)	0.0205 (10)	0.0115 (9)	−0.0013 (7)	0.0020 (7)	0.0037 (8)
C2	0.0127 (9)	0.0200 (10)	0.0125 (9)	−0.0018 (7)	0.0043 (7)	0.0022 (8)
C3	0.0174 (10)	0.0232 (11)	0.0190 (11)	0.0020 (8)	0.0056 (8)	−0.0012 (9)
C4	0.0157 (10)	0.0415 (14)	0.0202 (11)	0.0039 (9)	0.0073 (8)	−0.0029 (10)
C5	0.0150 (10)	0.0476 (15)	0.0190 (11)	−0.0069 (10)	0.0094 (8)	0.0013 (10)
C6	0.0151 (10)	0.0315 (12)	0.0145 (10)	−0.0068 (9)	0.0024 (8)	0.0056 (9)
C7	0.0245 (11)	0.0354 (14)	0.0196 (11)	−0.0144 (10)	0.0012 (9)	0.0093 (10)
C8	0.0333 (13)	0.0218 (11)	0.0222 (12)	−0.0125 (10)	−0.0029 (10)	0.0082 (9)
C9	0.0296 (12)	0.0175 (10)	0.0157 (10)	−0.0024 (9)	−0.0021 (9)	0.0013 (8)
C10	0.0449 (15)	0.0219 (12)	0.0286 (13)	0.0047 (11)	0.0038 (11)	−0.0028 (10)
C11	0.0173 (9)	0.0198 (10)	0.0145 (10)	−0.0015 (8)	0.0039 (8)	0.0043 (8)
C12	0.0174 (10)	0.0181 (10)	0.0152 (10)	−0.0014 (8)	0.0039 (8)	0.0033 (8)
C13	0.0255 (11)	0.0185 (10)	0.0173 (10)	0.0017 (8)	0.0039 (9)	0.0019 (8)
C14	0.0363 (13)	0.0183 (11)	0.0201 (11)	−0.0044 (9)	0.0034 (10)	0.0015 (9)
C15	0.0342 (13)	0.0251 (12)	0.0222 (12)	−0.0117 (10)	0.0064 (10)	0.0048 (9)
C16	0.0212 (10)	0.0288 (12)	0.0150 (10)	−0.0074 (9)	0.0039 (8)	0.0051 (9)
C17	0.0233 (11)	0.0429 (15)	0.0236 (12)	−0.0098 (10)	0.0107 (10)	0.0055 (11)
C18	0.0227 (11)	0.0450 (15)	0.0234 (12)	−0.0038 (10)	0.0145 (10)	0.0028 (11)
C19	0.0192 (10)	0.0338 (13)	0.0162 (11)	0.0014 (9)	0.0069 (8)	0.0020 (9)
C20	0.0282 (12)	0.0333 (13)	0.0263 (13)	0.0037 (10)	0.0148 (10)	0.0003 (10)

### Geometric parameters (Å, °)

**Table:**

La1—O2	2.3308 (15)	C4—C5	1.364 (4)
La1—O1^i^	2.4704 (15)	C4—H4	0.9500
La1—O1	2.4953 (15)	C5—C6	1.413 (4)
La1—O9	2.5553 (17)	C5—H5	0.9500
La1—O6	2.5759 (17)	C6—C7	1.422 (3)
La1—O3	2.5932 (16)	C7—C8	1.356 (4)
La1—O7	2.6267 (16)	C7—H7	0.9500
La1—O10	2.6365 (18)	C8—C9	1.404 (4)
La1—O4	2.6499 (16)	C8—H8	0.9500
O1—C2	1.340 (2)	C9—C10	1.482 (4)
O1—La1^i^	2.4704 (15)	C10—H10A	0.9800
O2—C12	1.312 (3)	C10—H10B	0.9800
O3—N3	1.282 (3)	C10—H10C	0.9800
O4—N3	1.269 (3)	C11—C16	1.409 (3)
O5—N3	1.214 (3)	C11—C12	1.431 (3)
O6—N4	1.271 (3)	C12—C13	1.395 (3)
O7—N4	1.271 (3)	C13—C14	1.403 (3)
O8—N4	1.222 (3)	C13—H13	0.9500
O9—N5	1.268 (3)	C14—C15	1.373 (4)
O10—N5	1.276 (3)	C14—H14	0.9500
O11—N5	1.212 (3)	C15—C16	1.409 (4)
N1—C9	1.332 (3)	C15—H15	0.9500
N1—C1	1.374 (3)	C16—C17	1.416 (3)
N1—H1	0.875 (10)	C17—C18	1.369 (4)
N2—C19	1.332 (3)	C17—H17	0.9500
N2—C11	1.369 (3)	C18—C19	1.410 (3)
N2—H2	0.873 (10)	C18—H18	0.9500
C1—C6	1.407 (3)	C19—C20	1.489 (3)
C1—C2	1.414 (3)	C20—H20A	0.9800
C2—C3	1.384 (3)	C20—H20B	0.9800
C3—C4	1.408 (3)	C20—H20C	0.9800
C3—H3	0.9500		
			
O2—La1—O1^i^	147.09 (5)	O1—C2—C3	123.8 (2)
O2—La1—O1	85.68 (5)	O1—C2—C1	118.82 (19)
O1^i^—La1—O1	66.36 (6)	C3—C2—C1	117.34 (19)
O2—La1—O9	79.43 (5)	C2—C3—C4	121.2 (2)
O1^i^—La1—O9	105.44 (5)	C2—C3—H3	119.4
O1—La1—O9	131.04 (5)	C4—C3—H3	119.4
O2—La1—O6	90.03 (6)	C5—C4—C3	121.3 (2)
O1^i^—La1—O6	122.78 (5)	C5—C4—H4	119.4
O1—La1—O6	152.87 (5)	C3—C4—H4	119.4
O9—La1—O6	73.96 (6)	C4—C5—C6	119.5 (2)
O2—La1—O3	71.61 (5)	C4—C5—H5	120.2
O1^i^—La1—O3	118.10 (5)	C6—C5—H5	120.2
O1—La1—O3	81.59 (5)	C1—C6—C5	118.8 (2)
O9—La1—O3	134.40 (6)	C1—C6—C7	117.2 (2)
O6—La1—O3	71.66 (6)	C5—C6—C7	124.0 (2)
O2—La1—O7	136.33 (5)	C8—C7—C6	120.4 (2)
O1^i^—La1—O7	74.72 (5)	C8—C7—H7	119.8
O1—La1—O7	137.50 (5)	C6—C7—H7	119.8
O9—La1—O7	74.71 (6)	C7—C8—C9	121.5 (2)
O6—La1—O7	49.33 (5)	C7—C8—H8	119.3
O3—La1—O7	103.20 (5)	C9—C8—H8	119.3
O2—La1—O10	76.14 (6)	N1—C9—C8	117.6 (2)
O1^i^—La1—O10	82.77 (5)	N1—C9—C10	118.0 (2)
O1—La1—O10	81.90 (5)	C8—C9—C10	124.4 (2)
O9—La1—O10	49.32 (6)	C9—C10—H10A	109.5
O6—La1—O10	122.99 (6)	C9—C10—H10B	109.5
O3—La1—O10	144.64 (6)	H10A—C10—H10B	109.5
O7—La1—O10	110.09 (6)	C9—C10—H10C	109.5
O2—La1—O4	120.22 (5)	H10A—C10—H10C	109.5
O1^i^—La1—O4	74.90 (5)	H10B—C10—H10C	109.5
O1—La1—O4	82.87 (5)	N2—C11—C16	119.0 (2)
O9—La1—O4	144.12 (6)	N2—C11—C12	118.05 (19)
O6—La1—O4	76.23 (6)	C16—C11—C12	122.9 (2)
O3—La1—O4	48.71 (5)	O2—C12—C13	125.5 (2)
O7—La1—O4	70.83 (5)	O2—C12—C11	118.66 (19)
O10—La1—O4	156.62 (5)	C13—C12—C11	115.9 (2)
C2—O1—La1^i^	123.42 (12)	C12—C13—C14	121.5 (2)
C2—O1—La1	122.24 (12)	C12—C13—H13	119.3
La1^i^—O1—La1	113.64 (6)	C14—C13—H13	119.3
C12—O2—La1	163.94 (14)	C15—C14—C13	121.9 (2)
N3—O3—La1	98.69 (13)	C15—C14—H14	119.0
N3—O4—La1	96.33 (12)	C13—C14—H14	119.0
N4—O6—La1	97.65 (13)	C14—C15—C16	119.4 (2)
N4—O7—La1	95.21 (12)	C14—C15—H15	120.3
N5—O9—La1	97.36 (13)	C16—C15—H15	120.3
N5—O10—La1	93.33 (13)	C11—C16—C15	118.4 (2)
C9—N1—C1	124.0 (2)	C11—C16—C17	117.0 (2)
C9—N1—H1	116.9 (19)	C15—C16—C17	124.5 (2)
C1—N1—H1	119.0 (19)	C18—C17—C16	121.5 (2)
C19—N2—C11	124.5 (2)	C18—C17—H17	119.3
C19—N2—H2	118.1 (18)	C16—C17—H17	119.3
C11—N2—H2	117.4 (18)	C17—C18—C19	119.8 (2)
O5—N3—O4	122.9 (2)	C17—C18—H18	120.1
O5—N3—O3	121.1 (2)	C19—C18—H18	120.1
O4—N3—O3	115.96 (18)	N2—C19—C18	118.1 (2)
O8—N4—O6	121.1 (2)	N2—C19—C20	118.2 (2)
O8—N4—O7	121.5 (2)	C18—C19—C20	123.7 (2)
O6—N4—O7	117.34 (18)	C19—C20—H20A	109.5
O11—N5—O9	121.4 (2)	C19—C20—H20B	109.5
O11—N5—O10	121.7 (3)	H20A—C20—H20B	109.5
O9—N5—O10	116.9 (2)	C19—C20—H20C	109.5
N1—C1—C6	119.2 (2)	H20A—C20—H20C	109.5
N1—C1—C2	119.01 (18)	H20B—C20—H20C	109.5
C6—C1—C2	121.8 (2)		
			
O2—La1—O1—C2	27.09 (15)	O6—La1—O10—N5	2.99 (16)
O1^i^—La1—O1—C2	−170.71 (18)	O3—La1—O10—N5	−102.47 (16)
O9—La1—O1—C2	99.05 (15)	O7—La1—O10—N5	56.84 (15)
O6—La1—O1—C2	−54.5 (2)	O4—La1—O10—N5	144.81 (15)
O3—La1—O1—C2	−44.94 (15)	La1—O4—N3—O5	−173.9 (2)
O7—La1—O1—C2	−145.33 (14)	La1—O4—N3—O3	5.5 (2)
O10—La1—O1—C2	103.68 (15)	La1—O3—N3—O5	173.8 (2)
O4—La1—O1—C2	−94.12 (15)	La1—O3—N3—O4	−5.7 (2)
O2—La1—O1—La1^i^	−162.20 (7)	La1—O6—N4—O8	172.3 (2)
O1^i^—La1—O1—La1^i^	0.0	La1—O6—N4—O7	−7.0 (2)
O9—La1—O1—La1^i^	−90.24 (8)	La1—O7—N4—O8	−172.5 (2)
O6—La1—O1—La1^i^	116.21 (11)	La1—O7—N4—O6	6.8 (2)
O3—La1—O1—La1^i^	125.77 (6)	La1—O9—N5—O11	−160.3 (2)
O7—La1—O1—La1^i^	25.38 (10)	La1—O9—N5—O10	18.1 (2)
O10—La1—O1—La1^i^	−85.61 (7)	La1—O10—N5—O11	161.0 (3)
O4—La1—O1—La1^i^	76.59 (6)	La1—O10—N5—O9	−17.4 (2)
O1^i^—La1—O2—C12	−2.5 (6)	C9—N1—C1—C6	−2.5 (3)
O1—La1—O2—C12	28.6 (5)	C9—N1—C1—C2	177.0 (2)
O9—La1—O2—C12	−104.6 (5)	La1^i^—O1—C2—C3	−79.0 (2)
O6—La1—O2—C12	−178.2 (5)	La1—O1—C2—C3	90.8 (2)
O3—La1—O2—C12	111.1 (5)	La1^i^—O1—C2—C1	100.81 (19)
O7—La1—O2—C12	−158.8 (5)	La1—O1—C2—C1	−89.4 (2)
O10—La1—O2—C12	−54.1 (5)	N1—C1—C2—O1	−3.6 (3)
O4—La1—O2—C12	107.7 (5)	C6—C1—C2—O1	175.98 (19)
O2—La1—O3—N3	−172.85 (14)	N1—C1—C2—C3	176.21 (19)
O1^i^—La1—O3—N3	−27.22 (14)	C6—C1—C2—C3	−4.2 (3)
O1—La1—O3—N3	−84.63 (13)	O1—C2—C3—C4	−178.2 (2)
O9—La1—O3—N3	133.73 (13)	C1—C2—C3—C4	2.1 (3)
O6—La1—O3—N3	90.79 (13)	C2—C3—C4—C5	1.0 (4)
O7—La1—O3—N3	52.32 (14)	C3—C4—C5—C6	−2.0 (4)
O10—La1—O3—N3	−147.60 (12)	N1—C1—C6—C5	−177.14 (19)
O4—La1—O3—N3	3.24 (12)	C2—C1—C6—C5	3.3 (3)
O2—La1—O4—N3	1.05 (15)	N1—C1—C6—C7	3.6 (3)
O1^i^—La1—O4—N3	149.16 (13)	C2—C1—C6—C7	−176.0 (2)
O1—La1—O4—N3	81.80 (13)	C4—C5—C6—C1	−0.1 (3)
O9—La1—O4—N3	−115.25 (14)	C4—C5—C6—C7	179.1 (2)
O6—La1—O4—N3	−80.79 (13)	C1—C6—C7—C8	−1.8 (3)
O3—La1—O4—N3	−3.25 (12)	C5—C6—C7—C8	178.9 (2)
O7—La1—O4—N3	−132.09 (14)	C6—C7—C8—C9	−1.1 (4)
O10—La1—O4—N3	131.47 (16)	C1—N1—C9—C8	−0.5 (3)
O2—La1—O6—N4	166.36 (14)	C1—N1—C9—C10	179.4 (2)
O1^i^—La1—O6—N4	−10.92 (16)	C7—C8—C9—N1	2.3 (3)
O1—La1—O6—N4	−113.08 (15)	C7—C8—C9—C10	−177.6 (2)
O9—La1—O6—N4	87.38 (14)	C19—N2—C11—C16	−0.9 (3)
O3—La1—O6—N4	−123.06 (15)	C19—N2—C11—C12	178.4 (2)
O7—La1—O6—N4	3.96 (12)	La1—O2—C12—C13	75.9 (6)
O10—La1—O6—N4	92.94 (15)	La1—O2—C12—C11	−103.9 (5)
O4—La1—O6—N4	−72.44 (14)	N2—C11—C12—O2	−0.3 (3)
O2—La1—O7—N4	−29.91 (17)	C16—C11—C12—O2	178.99 (19)
O1^i^—La1—O7—N4	163.12 (14)	N2—C11—C12—C13	179.88 (19)
O1—La1—O7—N4	139.10 (12)	C16—C11—C12—C13	−0.8 (3)
O9—La1—O7—N4	−85.73 (14)	O2—C12—C13—C14	179.9 (2)
O6—La1—O7—N4	−3.94 (13)	C11—C12—C13—C14	−0.3 (3)
O3—La1—O7—N4	47.18 (14)	C12—C13—C14—C15	0.8 (4)
O10—La1—O7—N4	−120.70 (13)	C13—C14—C15—C16	−0.1 (4)
O4—La1—O7—N4	84.13 (13)	N2—C11—C16—C15	−179.2 (2)
O2—La1—O9—N5	70.58 (14)	C12—C11—C16—C15	1.4 (3)
O1^i^—La1—O9—N5	−75.98 (14)	N2—C11—C16—C17	2.4 (3)
O1—La1—O9—N5	−4.11 (16)	C12—C11—C16—C17	−176.9 (2)
O6—La1—O9—N5	163.69 (15)	C14—C15—C16—C11	−1.0 (3)
O3—La1—O9—N5	121.40 (14)	C14—C15—C16—C17	177.2 (2)
O7—La1—O9—N5	−144.95 (14)	C11—C16—C17—C18	−2.3 (4)
O10—La1—O9—N5	−10.16 (13)	C15—C16—C17—C18	179.4 (2)
O4—La1—O9—N5	−161.43 (13)	C16—C17—C18—C19	0.7 (4)
O2—La1—O10—N5	−77.84 (14)	C11—N2—C19—C18	−0.8 (3)
O1^i^—La1—O10—N5	127.61 (14)	C11—N2—C19—C20	178.9 (2)
O1—La1—O10—N5	−165.36 (14)	C17—C18—C19—N2	0.9 (4)
O9—La1—O10—N5	10.04 (13)	C17—C18—C19—C20	−178.8 (2)

Symmetry codes: (i) −*x*+1, −*y*+1, −*z*+1.

### Hydrogen-bond geometry (Å, °)

**Table:**

*D*—H···*A*	*D*—H	H···*A*	*D*···*A*	*D*—H···*A*
N1—H1···O4^i^	0.88 (1)	2.39 (2)	3.115 (3)	140 (2)
N2—H2···O3	0.87 (1)	2.08 (1)	2.950 (2)	173 (2)

Symmetry codes: (i) −*x*+1, −*y*+1, −*z*+1.
